# Shugoshins: Tension-Sensitive Pericentromeric Adaptors Safeguarding Chromosome Segregation

**DOI:** 10.1128/MCB.01176-14

**Published:** 2015-01-23

**Authors:** Adele L. Marston

**Affiliations:** The Wellcome Trust Centre for Cell Biology, School of Biological Sciences, Edinburgh, United Kingdom

## Abstract

The shugoshin/Mei-S332 family are proteins that associate with the chromosomal region surrounding the centromere (the pericentromere) and that play multiple and distinct roles in ensuring the accuracy of chromosome segregation during both mitosis and meiosis. The underlying role of shugoshins appears to be to serve as pericentromeric adaptor proteins that recruit several different effectors to this region of the chromosome to regulate processes critical for chromosome segregation. Crucially, shugoshins undergo changes in their localization in response to the tension that is exerted on sister chromosomes by the forces of the spindle that will pull them apart. This has led to the idea that shugoshins provide a platform for activities required at the pericentromere only when sister chromosomes lack tension. Conversely, disassembly of the shugoshin pericentromeric platform may provide a signal that sister chromosomes are under tension. Here the functions and regulation of these important tension-sensitive pericentromeric proteins are discussed.

## CHROMOSOME SEGREGATION DURING MITOSIS AND MEIOSIS

### Mitosis.

During the somatic cell cycle, the genome is duplicated, and then one copy of each chromosome is segregated into two new daughter cells with genome content identical to that of the mother cell from which they originated. To achieve this equal partitioning of the chromosomes, the newly duplicated chromosomes are linked together as they replicate in S phase by the establishment of a protein complex, known as cohesin (reviewed in reference 1).

**FIG 1:**
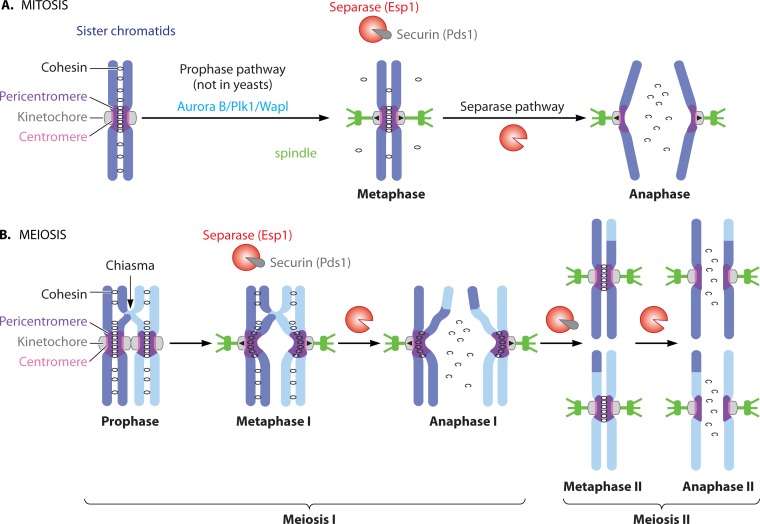
Chromosome segregation during mitosis and meiosis. (A) Pathways to cohesin loss during mitosis. During prophase, cohesin dissociates from chromosome arms in a manner dependent on Wapl. Once chromosomes are properly bioriented, separase is activated, and this cleaves the remaining cohesin, triggering chromosome segregation. (B) Spatial loss of cohesin during meiosis. Separase-dependent cleavage of cohesin on chromosome arms during meiosis I leads to the resolution of chiasmata and the segregation of homologous chromosomes. Protected pericentromeric cohesin allows sister chromatids to biorient during meiosis II, resulting in separase activation and cleavage of this cohesin.

During mitosis, replicated and cohered sister chromatids attach to microtubules of the mitotic spindle through the kinetochores that are assembled at a specific locus on each chromosome, called the centromere (Fig. 1A). Kinetochores of sister chromatids must attach to microtubules that grow from opposite poles to ensure their later segregation to different daughter cells. In this state, known as sister kinetochore biorientation, cohesin between sister chromatids resists microtubule pulling forces, thereby generating tension that serves as a signal that chromosomes are properly attached to microtubules (reviewed in reference 2). Only once all the chromosomes have achieved sister kinetochore biorientation, does a protease, separase, become active and cleave cohesin, triggering the segregation of sister chromatids to opposite poles (reviewed in reference 1). While separase-dependent cohesin cleavage is the universal trigger for chromosome segregation, in mammals the bulk of cohesin is removed from chromosomes during prophase through a nonproteolytic mechanism. This so-called “prophase” pathway removes the majority of cohesin from chromosome arms but not pericentromeres, where cohesin is preserved until separase activation at anaphase onset.

Cohesin is a ring-shaped complex thought to hold sister chromosomes together by embracing them (reviewed in reference 3). The core cohesin complex consists of two structural maintenance of chromosome (Smc) subunits, Smc1 and Smc3, an Scc3/stromal antigen (SA/STAG) subunit and an α-kleisin subunit (Scc1/Rad21/Mcd1 in mitotic cells), which together form a ring that is thought to encompass the two daughter DNA strands. Cleavage of the α-kleisin subunit by separase opens the ring and triggers chromosome separation. Cohesin association with chromosomes is the result of a balance between cohesin loading and unloading (reviewed in reference 1). Prior to S phase, the Scc2/Scc4 (also called Nipped-B-like [NIPBL]/Mau4) cohesin loader enables the association of cohesin with chromosomes (4–9). This loading reaction is thought to involve opening of the “hinge” interface between Smc1 and Smc3 in a manner dependent on hydrolysis of ATP by the Smc proteins (10–14). Core cohesin recruits the accessory subunit Pds5 (Pds5A/B in vertebrates) through the α-kleisin subunit (15–18). Pds5 recruitment enables the association of Wapl/Rad61 which promotes cohesin release, probably by opening the Smc3-Scc1 interface (17, 19–21). Therefore, before S phase, cohesin turns over rapidly on chromosomes due to the competition between the loading activity of Scc2/Scc4 and the destabilizing activity of Wapl. Stable association of cohesin with chromosomes, and establishment of cohesion, occurs only upon acetylation of its Smc3 subunit by the Eco1 acetyltransferase (ESCO1 and ESCO2 in vertebrates) during DNA replication (22–26). This acetylation, proximal to the Smc3-Scc1 interface is thought to lock the cohesin ring shut by making it insensitive to the destabilizing ability of Wapl. In yeasts, where a prophase pathway of cohesin removal is not apparent, Smc3 acetylation appears to be sufficient to counteract the destabilizing activity of Wapl. In mammals, however, Smc3 acetylation recruits the sororin protein, and this is essential to counteract Wapl activity and stabilize cohesin (27–30). Sororin competes with Wapl for binding to Pds5, suggesting that sororin stabilizes cohesin by dislodging Wapl from cohesin (27).

During prophase, sororin is phosphorylated by the cyclin-dependent kinase (CDK) and Aurora B kinases, which disrupts its interaction with Pds5, enabling Wapl-dependent removal of cohesin from chromosome arms (27, 28, 31–33). Importantly, it is only the pool of cohesin that has been acetylated that requires sororin phosphorylation for its removal (32). The Plk1 kinase also promotes cohesin removal during prophase (34–37) by phosphorylating the SA2 cohesin subunit, though the mechanism by which this triggers cohesin loss remains unclear (32, 38).

### Meiosis.

To generate gametes, the process of chromosome segregation must be modified so that the genome content is reduced by half (1, 39). In a specialized cell division, called meiosis, chromosomes are duplicated and then undergo two consecutive chromosome segregation events, known as meiosis I and meiosis II (Fig. 1B) (reviewed in references 1 and 39). During meiosis I, the paternal and maternal chromosomes (known as homologs) are segregated (reductional division), while in meiosis II, the sister chromatids are segregated (equational division), similar to mitosis. Uniquely in meiosis I, kinetochores of sister chromatids attach to microtubules of the same pole, rather than opposite poles, which is called sister kinetochore monoorientation. Additionally, the homologs are now linked, usually as a result of meiotic recombination that generates sites of reciprocal exchange between the homologs, known as chiasmata. As in mitosis, sister chromatids are held together by cohesin complexes, which are modified to perform specialized roles during meiosis: notably, in many organisms, the Scc1 α-kleisin subunit is replaced by its meiosis-specific homolog, Rec8, and this substitution is essential for many meiosis-specific functions of cohesin (reviewed in reference 39; see below). Chiasmata are held in place owing to distal sister chromatid cohesion on chromosome arms enabling homologs to align on the meiosis I spindle. Kinetochore monorientation and chiasmata together ensure that homologs, rather than sister chromatids, are bioriented during meiosis I. Once homolog biorientation has occurred, separase becomes active. In contrast to mitosis, separase cleaves Rec8 only on chromosome arms, while Rec8 in centromeric regions is protected from separase activity during meiosis I by the presence of shugoshin. Protection of Rec8 and, thereby, the maintenance of centromeric cohesin is important, as it allows sister chromatids to form bipolar attachments to the meiosis II spindle. Only upon biorientation of sister chromatids during meiosis II does separase become active again and cleave residual pericentromeric cohesin. It is owing to their requirement for the protection of centromeric cohesin during meiosis I that shugoshins were first identified.

## DISCOVERY OF SHUGOSHINS

**FIG 2:**
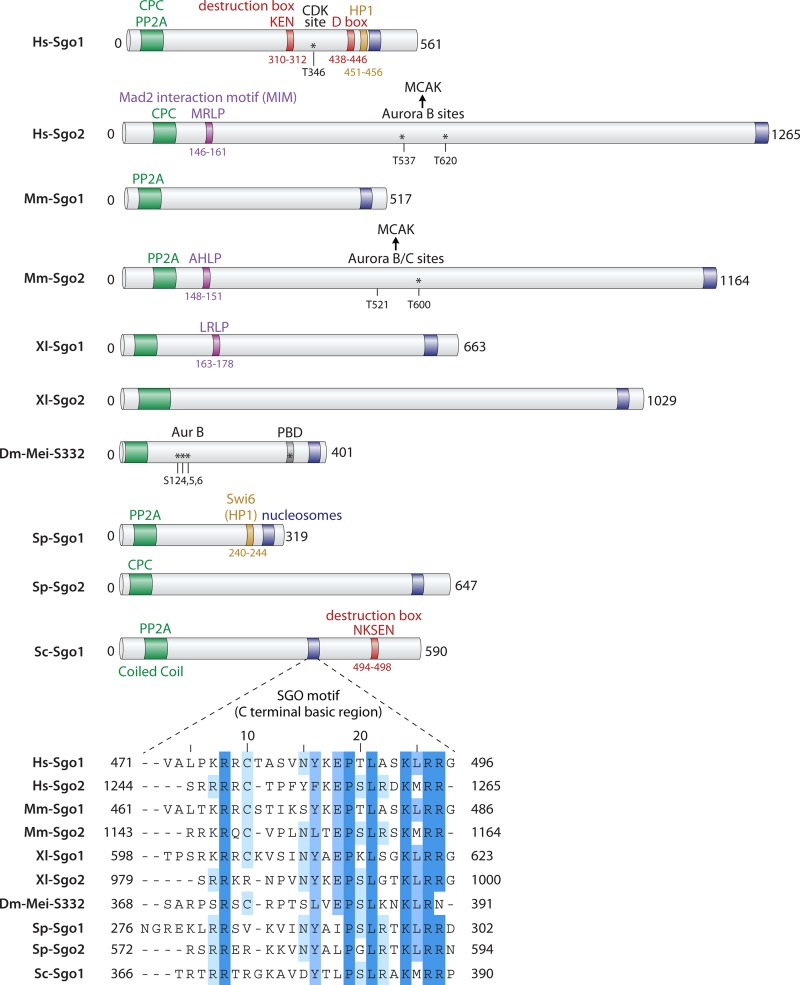
Organization of shugoshin proteins. Schematic diagram illustrating the key features of shugoshin proteins in organisms discussed in this minireview. Shugoshins carry a conserved basic “SGO motif” near the C terminus and a coiled-coil domain close to the N terminus, but they are otherwise relatively divergent. The coiled-coil domain has been found to enable shugoshin dimerization and association with PP2A and/or CPC; the SGO motif is required for the interaction with phosphorylated histone H2A. Motifs found to be important for association with HP1 or Mad2 (amino acid sequences MRLP, AHLP, and LRLP) or that are required for degradation (K box, D box, and amino acid sequence NKSEN) are also shown. The sites of regulatory phosphorylation events are also indicated. For details, see the text. PBD, Polo-binding domain.

The retention of cohesin in the pericentromeric region until meiosis II predicted the existence of a “protector” of pericentromeric cohesion during meiosis I. A Drosophila mutant, *mei-S332*, was identified; this mutant appeared to lose pericentromeric cohesion prematurely during meiosis (40). Mei-S332 localizes at centromeres during meiosis until anaphase II, the time when cohesion is lost, as predicted for a protector of centromeric cohesion (41). However, at that point in time, counterparts of Mei-S332 were not identified in other organisms, so it was unclear whether the cohesion protector was conserved. A few years later, screens carried out in fission yeast Schizosaccharomyces pombe and budding yeast Saccharomyces cerevisiae identified genes required for protection of meiotic cohesion in these organisms (42–45). This defined a family of proteins, called “shugoshins” (meaning guardian of the genome in Japanese), of which Drosophila Mei-S332 turned out to be a distant relative (43). Subsequent work has revealed that shugoshins have roles at the pericentromere in addition to the protection of centromeric cohesion during meiosis. Though the mechanism is distinct, shugoshins also prevent cohesion loss at the centromere due to the effects of the prophase pathway during mammalian mitosis. Shugoshins additionally promote biorientation of sister chromatids during mitosis. Broadly, all these activities are carried out through the recruitment of effector protein complexes to the pericentromere, including protein phosphatase 2A (PP2A) for cohesin protection or the chromosome passenger complex (CPC), MCAK (mitotic centromere-associated kinesin)/Kif2A kinesin motor, and condensin for sister kinetochore biorientation (Table 1; see below). While Drosophila and budding yeast have a single shugoshin protein, fission yeast, plants, and mammals have two (Fig. 2) (46). Some of these shugoshins are present only in meiotic cells, while others (e.g., budding yeast Sgo1, Drosophila Mei-S332) are also present in somatic cells. Shugoshins are relatively divergent except for a conserved C-terminal “SGO” motif and an N-terminal coiled-coil domain (Fig. 2). Consistently, there is some variation in the functions and interactions of shugoshins. The nomenclature is also not consistent with the described functions for shugoshins between species: for example, the meiotic cohesin protector in fission yeast is Sgo1, while this is the role of mouse Sgo2 (see below; summarized in Table 1). To avoid confusion, throughout this minireview, shugoshins are referred to with a prefix corresponding to the organism which they originate from (i.e., human Hs-Sgo [Hs stands for Homo sapiens], mouse Mm-Sgo [Mm stands for Mus musculus], frog Xl-Sgo [Xl stands for Xenopus laevis], fruit fly Dm-Mei-S332 [Dm stands for Drosophila melanogaster], fission yeast Sp-Sgo [Sp stands for Schizosaccharomyces pombe], and budding yeast Sc-Sgo [Sc stands for Saccharomyces cerevisiae]). (Note that mouse and human Sgo1 and Sgo2 have also been called Sgol1 and Sgol2, respectively.) Overall, I will argue that the common function of shugoshins is to serve as pericentromeric adaptor proteins, which have been tailored to perform in different contexts.

**TABLE 1:** Summary of functions for shugoshin proteins

Function	Species	Shugoshin(s)	Effector	Reference(s)
Protection of pericentromeric cohesion during meiosis I	Budding yeast	Sc-Sgo1	PP2A-Rts1	42–44
	Fission yeast	Sp-Sgo1	PP2A-Par1	43, 45
	Drosophila	Mei-S332	?	40
	Arabidopsis	At-Sgo1, At-Sgo2	?	47, 48
	Rice	Os-Sgo1	?	49
	Maize	Zm-Sgo1	?	50
	Mouse	Mm-Sgo2	PP2A-B′ (B56)	51, 52, 63
Protection of cohesion during mitosis	Xenopus	Xl-Sgo1	PP2A-B′ (B56γ)	17, 72
	Human	Hs-Sgo1	PP2A-B′ (B56)	65–71
Biorientation of sister chromatids during mitosis	Budding yeast	Sc-Sgo1	CPC	76, 89
			Condensin	76, 89
			PP2A-Rts1	89, 90
	Fission yeast	Sp-Sgo2	CPC	78, 87
	Xenopus	Xl-Sgo2	CPC^*a*^	73
			MCAK	
			PP2A-B56ε	
	Human	Hs-Sgo1	CPC	95
		Hs-Sgo2		
		Hs-Sgo2	MCAK	68
Spindle checkpoint silencing	Budding yeast	Sc-Sgo1	?	116
	Mouse	Mm-Sgo2	Mad2, PP2A	63
Biorientation of homologous chromosomes during meiosis I	Budding yeast	Sc-Sgo1	?	44, 88
	Mouse	Mm-Sgo2	?^*b*^	63

a Xl-Sgo2 affects the activation of the CPC, not its localization.

b Through inhibition of Aurora B/C kinases, though the relevant effector is unknown.

## FUNCTIONS OF SHUGOSHINS IN PROTECTING COHESION

### Mechanism of pericentromeric cohesin protection during meiosis I.

**FIG 3:**
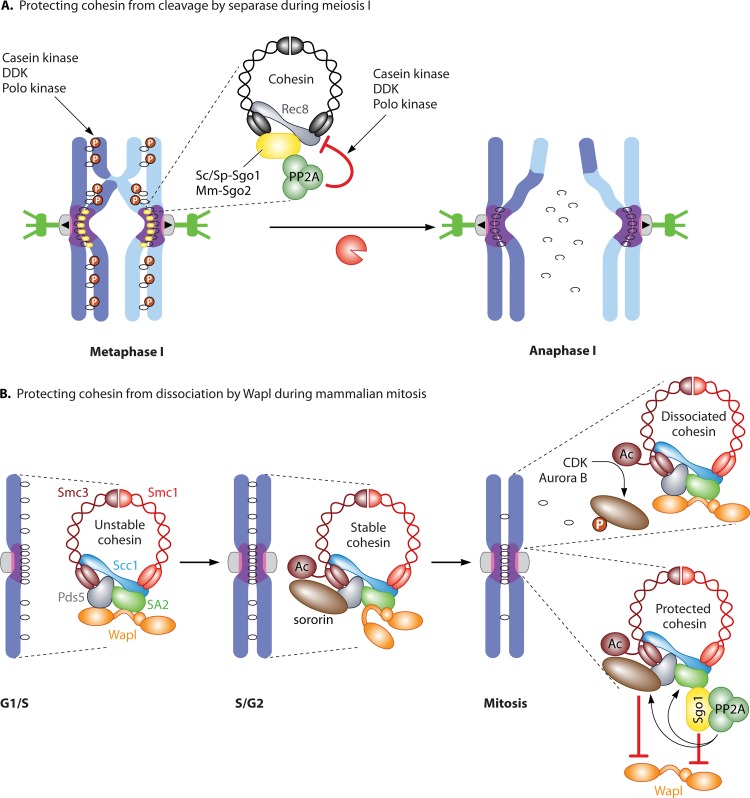
Protection of cohesin by shugoshins. (A) Mechanism of protecting pericentromeric cohesin from cleavage by separase during meiosis I. Shugoshin recruits PP2A, which prevents Rec8 phosphorylation, making it a poor substrate for separase-dependent cleavage. DDK, Dbf4-dependent kinase. (B) Mechanism of protecting pericentromeric cohesin from dissociation by Wapl during mammalian mitosis. In G_1_, Wapl can associate with cohesin and make it stable. After S phase, Eco1-dependent acetylation stabilizes cohesin in mammals by associating with sororin, which counteracts Wapl activity. During mitosis, CDK and Aurora B phosphorylate sororin, leading to its release from cohesin and making it susceptible to Wapl. At the pericentromere, shugoshin-PP2A protects cohesin from Wapl activity in two ways: preventing sororin phosphorylation and blocking Wapl binding to SA2.

The canonical role of shugoshins as protectors of pericentromere cohesion from separase activity during meiosis is well conserved and is known to require budding yeast Sc-Sgo1 (42–44), fission yeast Sp-Sgo1 (43, 45), Arabidopsis At-Sgo1 (At stands for Arabidopsis thaliana) and At-Sgo2 (47, 48), rice Os-Sgo1 (Os stands for Oryza sativa) (49), maize Zm-Sgo1 (Zm stands for *Zea mays*) (50), Drosophila Dm-Mei-S332 (40), and mouse Mm-Sgo2 (51, 52). Since the discovery of shugoshin in yeasts, the molecular mechanism underlying the protection of centromeric cohesin during meiosis has been worked out (Fig. 3A). During meiosis in many organisms, the Scc1/Rad21/Mcd1 α-kleisin is substituted by the meiosis-specific Rec8 kleisin (reviewed in reference 39). Rec8-containing cohesin performs multiple functions during meiosis that cannot be fulfilled by the Scc1 counterpart, including the maintenance of pericentromeric cohesion until meiosis II (53, 54). Rec8 phosphorylation is essential for its cleavage by separase (55–59), but at the pericentromere, shugoshins counteract Rec8 phosphorylation by recruiting a specific form of the protein phosphatase 2A, thereby preventing its cleavage (60–62). PP2A is a three-subunit complex comprised of a scaffold (A), regulatory (B), and catalytic subunit (C). Sc-Sgo1 and Hs-Sgo1 associate only with a specific holoenzyme containing the B′ regulatory subunit (60–62). PP2A-B′ is recruited to centromeres during meiosis by budding yeast Sc-Sgo1 and fission yeast Sp-Sgo1 and is required to protect cohesion (60–62). Similarly, recruitment of PP2A by mouse Mm-Sgo2 in oocytes is required for pericentromeric cohesin maintenance during meiosis I (63). Artificial tethering of fission yeast PP2A to chromosome arms prevents Rec8 cleavage along chromosomes, demonstrating that PP2A recruitment to the pericentromere by shugoshin is sufficient to prevent Rec8 cleavage (61). Similarly, artificial expression of Mm-Sgo1 in oocytes blocks the segregation of homologous chromosomes but only when it is capable of interacting with PP2A (62). Together these findings demonstrate a conserved role for Sgo-PP2A in rendering pericentromeric Rec8 resistant to separase activity during meiosis I.

At least in yeasts, the relevant substrate for PP2A in protecting cohesin from separase activity during meiosis is Rec8. Mutation of phosphorylated sites identified by mass spectrometry in budding or fission yeast Rec8 to alanine slowed or precluded Rec8 cleavage (57–59). Moreover, substitution of *in vivo*-phosphorylated residues in Sc-Rec8 with the phosphomimetic aspartate leads to its cleavage all along chromosomes during meiosis I, despite the presence of Sc-Sgo1–PP2A (57). The kinases that phosphorylate Rec8 to promote its cleavage show some redundancy as well as species specificity: casein kinase contributes in both budding and fission yeast, while Dbf4-dependent kinase (DDK) and Polo kinase are hitherto known to promote Rec8 cleavage only in budding yeast (56, 57, 59, 64). Overall, these studies demonstrate that PP2A-B′ recruitment by shugoshin prevents the separase-dependent cleavage of pericentromeric Rec8 during meiosis I by antagonizing its phosphorylation.

### Pericentromeric cohesin protection during mitosis in vertebrates.

Protection of pericentromeric cohesin from the prophase pathway (i.e., Wapl-destabilizing activity) is conferred by Hs-Sgo1 in human cell lines, though Hs-Sgo2 is present in mitotic cells (65–71). In Xenopus, Xl-Sgo1 also protects pericentromeric cohesin from the prophase pathway, though Xl-Sgo2 does not (17, 72, 73). Mice lacking *sgo2* are viable but infertile, suggesting that Mm-Sgo1 is the main Wapl antagonist in mouse somatic cells too (51). In budding yeast, the prophase pathway of cohesin removal does not seem to exist because cohesin levels and cohesion are maintained during mitosis in cells lacking Sc-Sgo1 (74–76). Similarly, although a fraction of cohesin dissociates from fission yeast chromosomes in early mitosis (77), there is no evidence that shugoshins regulate cohesion loss during mitosis in this organism (78).

Hs-Sgo1 protects pericentromeric cohesin from the prophase pathway in human cells, thereby restricting removal of cohesin to chromosome arms during prophase (60, 61, 67) (Fig. 3B). The cohesin protection capability of Hs-Sgo1 is activated by cyclin-dependent kinase (CDK) phosphorylation on T346, which enables Sgo1 binding to cohesin, bringing with it PP2A (33). The binding of Hs-Sgo1 to cohesin is essential for its cohesin protection function (79). Three activities of the Sgo1-PP2A complex contribute to the protection of pericentromeric cohesion during mammalian mitosis (Fig. 3B). First, Hs-Sgo1-PP2A ensures sororin maintenance at the centromere. Sororin is concentrated at centromeres in an Hs-Sgo1-dependent manner (27) and is a critical downstream target of Hs-Sgo1-PP2A in cohesin protection. Hs-Sgo1-PP2A counteracts Aurora B and CDK to maintain sororin in the dephosphorylated state, preserving its association with Pds5 and thereby counteracting cohesin removal by Wapl (31–33). Therefore, CDK both promotes and prevents cohesin loss during prophase through phosphorylation of sororin and Hs-Sgo1, respectively (27, 28, 31–33). Second, Hs-Sgo1-PP2A prevents cohesin dissociation through SA2 dephosphorylation (38, 61, 69). Dephosphorylation of sororin and SA2 appear to independently promote cohesin protection since nonphosphorylatable sororin and SA2 mutants have additive effects on slowing cohesin dissociation (32). Third, a recent crystal structure of the SA2 cohesin subunit in complex with an Scc1 fragment allowed the binding site for Hs-Sgo1 on cohesin to be mapped (80, 81). Interestingly, Hs-Sgo1 and Wapl were found to bind to the same interface, suggesting that Hs-Sgo1 directly counteracts the association of Wapl with cohesin (81).

Although all the available evidence provides a strong argument for collaboration between Sgo1 and PP2A in protecting pericentromeric cohesion from the prophase pathway during mammalian mitosis, the identity and regulation of the relevant pool of PP2A is still not completely clear. Depletion of Hs-Sgo2 has little effect on cohesion (65) but delocalizes PP2A from centromeres (61). Conversely, depletion of Hs-Sgo1 from human cells results in loss of cohesion without affecting PP2A localization at centromeres (61, 67). Instead, Hs-Sgo1 is required for PP2A association with cohesin (33). Together with the observation that depletion of the major PP2A scaffold subunit, Aα, results in loss of centromeric cohesion (61, 67), these findings argue that Hs-Sgo1-dependent recruitment of PP2A to cohesin is important for its protection, while centromeric PP2A is not (33). Hs-Sgo1 copurifies specifically with PP2A containing its B′ family of regulatory subunits (82, 83) from mitotic cells and binds directly through this subunit (62). Though depletion of the major Aα scaffold subunit causes loss of cohesion (61, 67), paradoxically, depletion of all B′ regulatory subunits does not (82, 83). Therefore, the composition and specific interactions of the PP2A enzyme complex responsible for cohesion protection during mammalian mitosis are unclear. Analysis of specific Hs-Sgo1/Hs-Sgo2 and PP2A subunit alleles that specifically interrupt the interactions between them could help resolve this important issue.

## FUNCTIONS OF SHUGOSHINS IN CHROMOSOME BIORIENTATION

Accurate segregation relies on a system to ensure that chromosomes attach to microtubules from opposite poles, known as chromosome biorientation. In mitosis, it is the identical sister chromatids that attach to microtubules from opposite poles and are said to be bioriented. However, during meiosis I only, sister kinetochores attach to microtubules from the same pole (termed monoorientation or coorientation), and it is homologous chromosomes that are bioriented instead. In both cases, geometrical constraints are likely to promote chromosome biorientation. However, geometry in itself is not sufficient for biorientation, and where this fails, the resultant lack of tension engages the “error correction” process, the major role of which appears to be to destabilize tension-less kinetochore-microtubule attachments (reviewed in reference 2). The key player in this process is the chromosome passenger complex (CPC) comprised of Aurora B, its centromere-targeting factor, survivin, together with inner centromere protein INCENP and borealin (84). As cells biorient chromosomes, it is critical that cell cycle progression is blocked so that cohesin is not cleaved before biorientation is complete. This is the role of the spindle assembly checkpoint (SAC), which prevents separase activation in the presence of improper kinetochore-microtubule interactions (reviewed in reference 85).

### Biorientation of sister chromatids during mitosis.

An elegant screen in budding yeast first identified *Sc-SGO1* as a gene required for the response to a lack of tension between kinetochores during mitosis (74). Budding yeast Sgo1 is required both to facilitate biorientation and to prevent cell cycle progression in response to the lack of tension that ensues when biorientation fails (74, 86). Subsequently, fission yeast Sp-Sgo2 and human Hs-Sgo2 have been found to play a similar role. Shugoshins appear to promote chromosome biorientation by enabling the centromeric localization of several factors important for biorientation. These factors include the CPC, chromosome-shaping complex, condensin, PP2A, SAC component Mad2, and the kinesin MCAK, each with distinct functions in orienting chromosomes (63, 66, 76, 78, 86–91).

### (i) Biorientation through error correction.

A major role of shugoshins that is likely to underlie their function in kinetochore biorientation is to serve as an adaptor for the CPC at the pericentromere. The CPC is known to destabilize kinetochore-microtubule interactions that lack tension, thereby allowing these errors to be corrected (reviewed in reference 2). The pericentromeric localization of the CPC is thought to be critical to allow access to substrates in the kinetochore, the phosphorylation of which destabilizes interactions with microtubules when kinetochores are not under tension (92, 93). However, in budding yeast, prevention of CPC localization to the inner kinetochore through all previously described pathways did not preclude chromosome biorientation, at least under normal circumstances (94). Shugoshins have been shown to affect CPC localization in several organisms, providing a possible explanation of how they facilitate biorientation and prevent cell cycle progression where it fails (17, 72, 76, 78, 87, 89, 95–97). Budding yeast Sc-Sgo1 is not required for the initial association of the CPC with the centromere, but it is important for its maintenance during mitosis, which could explain why the CPC, but not Sgo1, is essential for biorientation in an unperturbed cell cycle (76, 89, 96). In fission yeast, CDK-dependent phosphorylation of the Bir1/survivin CPC subunit enables its association with the pericentromere through binding to the coiled-coil region of Sp-Sgo2, and this interaction is important for chromosome biorientation (78, 87, 95). In human cells, CDK1 phosphorylates a different CPC subunit, borealin, allowing it to bind to the coiled-coil regions of Hs-Sgo1 and Hs-Sgo2 which both contribute to the pericentromere localization of the CPC (95). In fission yeast and human cells (but not budding yeast), the localization of the CPC at the pericentromere is also under the control of the Haspin kinase (98–100). Haspin is recruited to chromosomes through an interaction with the cohesin subunit Pds5, and once it is positioned, it phosphorylates histone H3 on threonine 3 (H3-T3-P) to provide a docking site for the CPC subunit survivin. Pericentromeric CPC enrichment is therefore the product of the intersection of the cohesin-proximal H3-T3-P mark and shugoshin localization in the pericentromere (which also depends on a histone mark: phosphorylation of histone H2A on S121 (H2A-S121-P) by kinetochore-localized Bub1; see below).

Another effector of Hs-Sgo2 that is relevant for chromosome biorientation is the kinesin MCAK/Kif2a, which is known to promote proper kinetochore-microtubule attachments by helping them to turn over and which is recruited to centromeres by Hs-Sgo2 (66, 68, 101–104). Interestingly, MCAK recruitment by Hs-Sgo2 depends on Aurora B-dependent phosphorylation of the central region of Hs-Sgo2 (68). In Xenopus, Xl-Sgo1 mediates the localization of the CPC, while Xl-Sgo2 rather promotes its activity toward MCAK (73). It is important to note, however, that the interaction between MCAK and Hs-Sgo2 does not appear to be essential for mitosis. Although mitotic defects have been observed upon Hs-Sgo2 knockdown by small interfering RNA (siRNA), this is not consistently the case, hinting that Hs-Sgo2 becomes important only in cell lines that are already compromised (65, 66, 68). Mice lacking Mm-Sgo2 consistently show no overt chromosome segregation defects, despite delocalization of MCAK from kinetochores (51). These findings suggest that chromosome biorientation in mammals is under robust control comprised of several redundant pathways.

### (ii) Biorientation through geometry.

In budding yeast, the chromosome-organizing complex condensin is a further effector of Sc-Sgo1 in promoting kinetochore biorientation (76, 89). Cells lacking condensin function show impaired tension sensing and biorientation (105). Condensin is highly enriched in the pericentromere (106) and is important to provide a proper chromatin structure to resist spindle forces (107). The enrichment of condensin in the pericentromere requires Sc-Sgo1 (76, 89), explaining the altered pericentromeric chromatin structure observed when Sc-Sgo1 is absent (108). Pericentromeric condensin serves two functions in biorientation. First, condensin confers a bias on sister kinetochores to be captured by microtubules from opposite poles (76). Second, condensin in the pericentromere facilitates the error correction process driven by the CPC (76, 89). Although further studies are needed to understand exactly how condensin contributes to biorientation, it seems likely that it provides a particular structure or geometry that orients sister kinetochores in a “back-to-back” orientation.

One unresolved issue is the involvement of PP2A-B′/Rts1 in condensin-dependent sister kinetochore biorientation. One study found that cells lacking *RTS1* showed a reduction in pericentromeric condensin and missegregated chromosomes during anaphase after being challenged with microtubule-depolymerizing drugs (89). Consistently, in human and Xenopus cells, a noncatalytic function of PP2A promotes condensin II (but not condensin I) association with chromosomes (109). However, in two different studies, cells lacking *RTS1* achieved sister kinetochore biorientation efficiently in metaphase-arrested cells (76, 90). A possible explanation to reconcile these findings is if PP2A-B′/Rts1 is dispensable for biorientation during metaphase but essential both to elicit a cell cycle delay where biorientation fails (see below) and to maintain robust kinetochore-microtubule attachments during anaphase. In support of this idea, the B′ PP2A regulatory subunits are reported to stabilize kinetochore-microtubule attachments in human cells (82). Alternatively, a different form of PP2A, associated with the alternate “B” subunit (called Cdc55 in budding yeast) might promote biorientation downstream of Sc-Sgo1 in budding yeast when the B′ subunit (Rts1) is absent. In accordance with this view, cells carrying versions of Sc-Sgo1 that cannot bind PP2A do show biorientation defects (76, 89, 90), and artificial recruitment of PP2A can rescue the biorientation defects of budding yeast cells lacking Sc-Sgo1 (89, 90). Exactly how PP2A contributes to chromosome biorientation is an important question to address in the future.

### Delaying the cell cycle.

In addition to governing the error correction process, the CPC also plays a central role in preventing cell cycle progression in response to a lack of tension, predominantly by generating unattached kinetochores that activate the SAC, thereby stabilizing securin and maintaining separase inhibition (110–112). In budding yeast, Sc-Sgo1 may also delay cell cycle progression in response to a lack of tension between kinetochores independently of its role in localizing the CPC to the centromere. Both PP2A-B′/Rts1 and the alternative PP2A-B/Cdc55 holoenzyme are thought to contribute to delaying the cell cycle in response to a lack of tension downstream of Sc-Sgo1 (62, 89, 113). Although the underlying mechanisms are unclear, PP2A-B/Cdc55 at least appears to act independently of the SAC and securin (113). PP2A-Cdc55 inhibits cohesin cleavage by antagonizing Polo kinase (Cdc5)-dependent phosphorylation of the Scc1 cohesin subunit (114) but may also prevent separase activity directly (113). These observations indicate that shugoshins could act at multiple levels to elicit the response to a lack of tension between kinetochores. Interestingly, the requirement for Sc-Sgo1 in sensing tension can be largely overridden by artificial recruitment of either the CPC (89, 94) or PP2A (89, 90). Whether PP2A and CPC collaborate in the response to tension or perform separate, redundant roles remains unclear, but the finding that the CPC maintains PP2A-Rts1 at centromeres in budding yeast meiosis hints at the former possibility (115).

In vertebrates, Hs-Sgo2 and Xl-Sgo1 interact directly with the SAC component Mad2 (65). This raises the possibility that shugoshins directly engage the SAC to delay the cell cycle in response to a lack of tension. However, as described above, Hs-Sgo2 does not seem to be essential for mitosis (65, 66, 68). Furthermore, in mouse oocytes, Mm-Sgo2 interaction with Mad2 appears to be important not to activate but to silence the SAC (see below) (63). Therefore, the importance of the Sgo2-Mad2 interaction in somatic cells requires further investigation. Similarly, in budding yeast, Sc-Sgo1 has also been proposed to promote SAC silencing, though the underlying mechanism is unknown (116).

### Biorientation of homologous chromosomes during meiosis I.

Shugoshins are also required for accurate chromosome segregation during meiosis I, a function that is likely unrelated to their role in cohesion protection. Budding yeast Sc-Sgo1 (44, 88), fission yeast Sp-Sgo2 (43), and mouse Mm-Sgo2 (63) have all been found to be required for accurate segregation of homologs. In mice, Mm-Sgo2 silences the SAC by binding to Mad2 and PP2A (63). In addition to Aurora B, oocytes express an alternative CPC catalytic subunit, Aurora C, and both kinases contribute to the meiotic functions of the CPC (117). Aurora B/C-dependent phosphorylation of Mm-Sgo2 enables its association with MCAK, which restricts the stretching of homologous chromosomes, presumably by modulating the pulling forces from microtubules (63). Curiously, Mm-Sgo2 also inhibits Aurora B/C kinases at kinetochores to allow stabilization of kinetochore-microtubule interactions (63). Therefore, at least in mouse oocytes, both inhibition of Aurora B/C and recruitment of MCAK by Mm-Sgo2 are thought to promote homolog biorientation. The finding that Aurora B/C kinases may be inhibited by Mm-Sgo2 contrasts with the idea that other shugoshins play a positive role in Aurora B recruitment to promote biorientation (see above). This suggests the intriguing possibility that inhibition of Aurora B/C kinases by Mm-Sgo2 represents part of a meiosis I-specific mechanism to allow the unique suppression of sister kinetochore biorientation.

## BUILDING THE PERICENTROMERIC SHUGOSHIN PLATFORM

**FIG 4:**
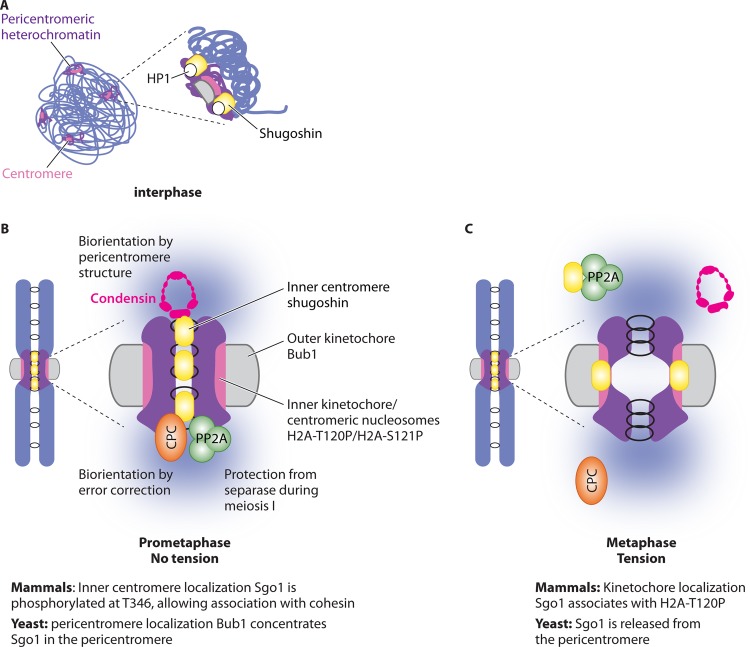
Localization of shugoshins. A generalized diagram is shown, indicating modes of shugoshin localization. (A) In mammalian cells, shugoshin associates with pericentromeric heterochromatin through an interaction with HP1 protein. (B) In mitosis in the absence of tension between sister kinetochores, shugoshin associates with the pericentromere (inner centromere), which in mammals depends on its phosphorylation at T346, allowing its association with cohesin. This allows localization of its effector proteins to the inner centromere/pericentromere. (C) Shugoshin localization at the pericentromere is sensitive to tension. In mammals, tension between sister kinetochores leads to dephosphorylation of T346 and relocation of Sgo1 to the kinetochore (associated with Bub1-dependent H2A-T120P). In budding yeast, Sgo1 dissociates from the pericentromeric chromatin when sister kinetochores are under tension, in a manner facilitated by PP2A-B′/Rts1. Disassembly of the pericentromeric platform is likely to stabilize the bioriented state by release of shugoshin effectors.

Shugoshins appear to play their critical roles in chromosome segregation when localized to the centromeric region. Mounting evidence indicates that this localization is due to the convergence of multiple control mechanisms that direct functionally important interactions with chromatin proteins, including heterochromatin protein 1 (HP1), nucleosomes, and cohesin in the centromere-proximal domain (Fig. 2). Furthermore, at least in human cells, these interactions appear to define functionally separable pools of shugoshin either overlapping with the kinetochore or with the so-called “inner centromere” (the chromatin domain on the interior face of the kinetochore between sister chromatids) (Fig. 4).

### Heterochromatin recruits shugoshin during interphase.

In most organisms, with the notable exception of budding yeast, centromeres are associated with pericentromeric heterochromatin, characterized by di- and trimethylation of histone H3 on lysine 9 (H3K9me2/3) to which HP1 binds (118). HP1 directly binds to shugoshin and contributes to its pericentromeric localization in fission yeast and mammals (Fig. 4A), though the relevance of this association for cohesin protection appears to differ between organisms (119, 120). In fission yeast, the Sp-Sgo1–HP1 interaction is functionally important because a mutant Sp-Sgo1 protein that loses the ability to associate with HP1 (called Swi6 in fission yeast) fails to properly protect cohesin during meiosis (119). The situation is less clear in mammalian cells (119–122) where there are three HP1 proteins, HP1α, HP1β, and HP1γ (123). During mitosis, however, most of the HP1 dissociates from chromatin owing to phosphorylation of histone H3 on the serine 10 residue (H3-pS10), which disrupts the H3K9Me2/3-chromodomain interaction (124, 125). Although one study reported that depletion of HP1α using siRNA led to delocalization of Hs-Sgo1 and a corresponding loss of centromere cohesion during mitosis (119), another study found no evidence for HP1 proteins in maintaining cohesion (122). Furthermore, inactivation of the Suv39h H3K9 methyltransferase did not lead to gross centromere cohesion defects in human cells (121), though the alternative Suv4-20h methyltransferase is important for proper cohesion (126). A later study used an Hs-Sgo1 mutant that specifically abrogated its binding to HP1 to demonstrate that this interaction is not essential for its centromeric localization or cohesion protection function during mitosis (120). Conversely, the centromeric localization of Hs-Sgo1 during interphase is dependent on its association with HP1, though the function of this pool of Hs-Sgo1 is not yet clear (120, 127). Overall, these findings suggest that HP1 may establish Hs-Sgo1 localization during interphase for optimal execution of its functions later in mitosis and/or that Hs-Sgo1 may have functions at the pericentromere during interphase.

### Bub1 kinase and cohesin direct shugoshin localization during mitosis.

The kinetochore-localized Bub1 kinase plays a central and conserved role in localizing shugoshins to the centromeric region, and this appears to be distinct from the HP1-mediated localization in interphase (43, 70, 75, 127–132). In addition to localizing shugoshin, Bub1 is also required for the SAC, which prevents separase activation in response to unattached kinetochores during mitosis (133). While the SAC function of Bub1 lies outside its kinase domain (134, 135), Bub1 kinase activity is essential for its shugoshin localization function (127, 131). Bub1 phosphorylates histone H2A at residue T120 (S121 in yeasts) to enable shugoshin enrichment in the centromeric region (79, 136). H2A-T120-P is localized within the kinetochore domain of the centromere in human cells and enables Hs-Sgo1 recruitment to the kinetochore but not the inner centromere (79). Instead, the inner centromere localization is mediated through the association of Hs-Sgo1 with cohesin, which depends on the prior phosphorylation of Hs-Sgo1 at T346 by CDK (33, 79). In the absence of Bub1/H2A-T120-P, Hs-Sgo1 localizes along chromosomes through its interaction with cohesin, resulting in the protection of arm cohesin from removal by the prophase pathway (33, 70, 129, 132). Therefore, it is the Hs-Sgo1–cohesin interaction, rather than its Bub1/H2A-T120-P-dependent kinetochore localization, that is functionally relevant in protecting cohesin from the prophase pathway of removal (79).

Whether the protection of cohesin from separase activity during meiosis also depends on cohesin-dependent localization of shugoshins, as opposed to Bub1/H2A-S121-P-dependent localization, remains to be determined. The fission yeast meiosis cohesin protector Sp-Sgo1 interacts with the meiosis-specific Sp-Rec8 cohesin subunit (43). In addition, Sc-Sgo1 requires cohesin for its localization during both mitosis and meiosis (75, 76). However, Sp-Bub1/H2A-S121-P also appears to be essential for the maintenance of pericentromeric cohesion until meiosis II (137), suggesting that both pathways of recruitment could play a role in cohesin protection during meiosis. Furthermore, although histone H2A phosphorylation is essential for the pericentromeric localization of shugoshin, it may not be sufficient and Bub1 may have other, as-yet-unidentified, relevant substrates. In budding yeast, the phosphomimetic H2A-S121D mutant supports Sc-Sgo1 pericentromeric localization, but only in the presence of Bub1 (96). In addition, further residues in yeast histone H2A as well as H3 are known to affect Sc-Sgo1 localization, suggesting that shugoshins may contact multiple components on the nucleosome (97, 138, 139).

The Mps1 kinase is also required for shugoshin localization to the pericentromere in budding yeast (91) and human cells (140). Mps1 is a central upstream kinase in the SAC (141) and is required for sensing tension and sister kinetochore biorientation (142). The requirement for Mps1 in shugoshin localization can be explained, in part, by its role in recruiting Bub1 to the kinetochore (141); however, Mps1 may also affect shugoshin localization, independently of Bub1 (91, 140).

### Roles of kinases and phosphatases in localizing shugoshin.

The localization of shugoshin and interactions with its effectors within the pericentromere is the product of a complex interplay between shugoshin and its effectors. It is reasonable to assume that cohesin together with the chromatin marks laid down by Hs-Bub1 provide the initial anchor for establishment of the pericentromeric shugoshin platform. Shugoshins in turn recruit PP2A and the CPC, which together with other local kinases influence the localization and activity of other proteins and each other. For example, Aurora B and shugoshins appear to have a reciprocal relationship in promoting the enrichment of each other in many organisms, while Polo kinase promotes shugoshin dissociation from the pericentromere (63, 66, 68, 73, 78, 95, 98, 130). Aurora B directly phosphorylates the Drosophila shugoshin Dm-Mei-S332 to ensure its stable association with chromosomes, while Polo kinase, though associated with its Polo-binding domain (PBD) and phosphorylation, promotes its dissociation during anaphase (143–145). In human culture cells, Hs-PP2A and Hs-Sgo1 are also mutually required for their localization. PP2A apparently promotes Hs-Sgo1 enrichment by counteracting the Sgo1-dissociating activity of Polo kinase (61, 67). Furthermore, in human cells, Aurora B-dependent phosphorylation of Sgo2 enables its binding to PP2A (68). Similarly, Aurora B is required for recruitment of PP2A by Sc-Sgo1, as well as cohesin protection, during budding yeast meiosis (115). Consistently, Aurora B has been shown to be required for cohesin protection in several organisms (68, 115, 144, 146). Overall, it can be concluded that the pericentromeric localization of the shugoshin platform is regulated by a complex network of feedback, the details of which are yet to be worked out.

## INACTIVATION OF SHUGOSHINS

After meiosis I, the protective function of shugoshins must be turned off to allow loss of centromeric cohesion and sister chromatid segregation during meiosis II. Similarly, during mitosis, shugoshin's protective and kinetochore-microtubule destabilizing capabilities would be expected to be inactivated to allow separase activation as well as its access to cohesin. So how is shugoshin regulated?

### Degradation of shugoshin.

A characteristic of shugoshin is its cell cycle-dependent degradation during anaphase, though there are organism-specific differences as to whether the degradation occurs in meiosis I or meiosis II (42–44, 66, 74, 78, 87). Shugoshin degradation seems to be dependent on the anaphase-promoting complex (APC), but the precise control of this degradation is not well understood (71, 90, 147). In human cells, two separate motifs (a “KEN” box and a “D box”) mediate Hs-Sgo1 destruction (147), while in budding yeast, a short sequence close to the C terminus was found to be important (90) (Fig. 2). Although shugoshin degradation will irreversibly inactivate it, this is probably not the primary event that cancels its pericentromeric functions. In support of this idea, ectopic Sp-Sgo1 cannot prevent cohesion loss during meiosis II (45), and expression of a nondegradable Sgo1 in human cells or budding yeast similarly does not prevent chromosome segregation (90, 147). Furthermore, although mutation of the Polo kinase-dependent sites in Drosophila Mei-S332 prevents its dissociation from centromeres, chromosome segregation occurs apparently normally during mitosis and meiosis (143, 145). Instead, tension-dependent changes in shugoshin localization together with direct inhibition of its effectors are together likely to counteract the effects of shugoshin.

### Tension-dependent relocalization of shugoshin.

The idea that tension between sister kinetochores could negatively regulate shugoshin is attractive since in both mitosis and meiosis, shugoshins would be expected to play their crucial functions at the pericentromere when sister kinetochores are not under tension. Indeed, tension-dependent relocalization of shugoshins has been observed during mitosis in budding yeast, mouse, and human cells as well as mouse oocytes (52, 79, 90, 96, 148) (Fig. 4). At least in mitotic cells, accumulating evidence suggests that relocalization of shugoshins could be central to the tension-sensing process (Fig. 4B and C). In general, intersister kinetochore tension appears to trigger shugoshin removal from the pericentromeric chromatin. How is the tension at sister kinetochores actually sensed by shugoshins? One possibility is that shugoshins are mechanically responsive to tension, so that a tension-induced structural change in shugoshins themselves or the pericentromeric chromatin triggers their dissociation. Alternatively, shugoshin relocalization may be chemically induced due to a shift in the proximity of phosphatases and kinases as tension separates kinetochores from the pericentromeric chromatin.

Support for the latter model has been obtained from studies of human cells and budding yeast. During metaphase of mitosis in human cells, Hs-Sgo1 relocates from the inner centromere, where it is bound to cohesin, toward the kinetochores, where it colocalizes with Bub1-mediated H2A-T120 phosphorylation (52, 79). Association of Hs-Sgo1 with cohesin requires its phosphorylation at T346, while its relocalization to kinetochores depends on its tension-dependent dephosphorylation at this residue (79). Since Hs-Sgo1 must be associated with cohesin for its protection from the prophase pathway (33), tension between sister kinetochores would be expected to turn off the Hs-Sgo1-dependent protection mechanism; however, in practice, pericentromeric cohesin is cleaved only after separase activation (79). How pericentromeric cohesin is spared from the effects of the prophase pathway after tension triggers Hs-Sgo1 removal remains to be seen.

In budding yeast, Sc-Sgo1 associates with the cohesin-rich pericentromere only when sister kinetochores are not under tension, though tension results in relocalization to the nucleus rather than the kinetochore in this organism (96). Interestingly, PP2A-B′ (Rts1) appears to negatively regulate Sc-Sgo1 association with the pericentromere, suggesting that dephosphorylation could be a general mechanism whereby tension-dependent shugoshin relocalization is triggered (96). In addition, continued Bub1 kinase activity is important to maintain the pericentromeric localization of Sc-Sgo1 (96). This suggested that tension-dependent separation of kinetochore-localized Bub1 and pericentromere-localized Sc-Sgo1–PP2A could trigger a phosphorylation-dependent switch, resulting in Sc-Sgo1 removal from the pericentromere (96). However, whether PP2A and Bub1 regulate the same pool of Sc-Sgo1 or share common substrates is not known. Therefore, further work is required to understand how tension is converted into phosphorylation-dependent relocalization of shugoshins. The importance of tension-dependent shugoshin removal from the pericentromere in signaling chromosome biorientation is demonstrated by the finding that tension-dependent Sc-Sgo1 removal also triggers the delocalization of its effectors (CPC, condensin, and PP2A) (96). This places disassembly of the pericentromeric shugoshin platform as a defining event in the sensing of sister kinetochore biorientation.

### Deprotection of cohesion during meiosis.

Does tension-dependent shugoshin relocation play a role in deprotecting cohesion during meiosis? In mouse meiosis, Mm-Sgo2 colocalizes with Rec8 at the inner centromere in metaphase I, but when sister chromatids become bioriented and under tension during meiosis II, Mm-Sgo2 moves toward the kinetochores and away from Rec8 (52, 148). It was hypothesized that this relocalization of Mm-Sgo2 might be sufficient to deprotect Rec8, thereby making it susceptible to cleavage upon separase activation. In support of this idea, fission yeast mutants that biorient sister chromatids during meiosis I fail to protect cohesion (128). In budding yeast, although loss of the sister kinetochore monoorientation complex, monopolin, does not result in the segregation of sister chromatids to opposite poles during meiosis I (53), only a fraction of sister kinetochores are bioriented in these cells. By preventing recombination in monopolin-deficient cells, the fraction of bioriented sister kinetochores can be increased, resulting in shugoshin delocalization and cohesin deprotection in some, but not all, cells during meiosis I (96). Therefore, while preventing tension between sister kinetochores appears to contribute to preventing precocious cohesin loss during meiosis I, other mechanisms must exist to safeguard pericentromeric cohesin. Because intersister kinetochore tension is not sufficient for cohesin deprotection during meiosis, other pathways must turn off the protection mechanism in meiosis II.

There is also evidence that shugoshin removal from the pericentromere is not actually required for cohesin deprotection during meiosis. In mouse oocytes, expression of stable cyclin A2 causes the precocious separation of sister chromatids during meiosis I without loss of Mm-Sgo2 from the centromere (149). Similarly, in budding yeast, expression of the meiosis II-specific cyclin Clb3 during meiosis I causes cohesin deprotection without delocalizing Sc-Sgo1 (150). Furthermore, examination of PP2A localization in mouse oocytes led to a different conclusion than the earlier studies where Rec8 and Mm-Sgo2 localization was examined (151). The authors observed colocalization of PP2A subunits with Rec8 during meiosis II, arguing that tension-dependent redistribution of Mm-Sgo2 is unlikely to be sufficient for deprotection of Rec8. Instead, I2PP2A, an PP2A inhibitor, colocalizes with Rec8 in meiosis II but not in meiosis I and is required for meiosis II chromosome segregation in mouse oocytes (151). How I2PP2A is itself regulated is an open question. Overall, these studies indicate that cohesin deprotection during meiosis II cannot be fully explained by tension-dependent relocation of shugoshin.

## SGO1: A TENSION-SENSITIVE PERICENTROMERIC ADAPTOR?

Since their discovery as protectors of pericentromeric cohesion during meiosis, shugoshins have emerged as central regulators of chromosome segregation. Shugoshins have been reported to perform several different functions (Table 1 and Fig. 2). What is the unifying feature of shugoshins that underlies these functions? The fundamental and universal property of shugoshins is their ability to recognize and associate with the pericentromere. The pericentromeric localization of shugoshins appears to be critical for their diverse functions. Pericentromeric shugoshin acts as a platform to position key effector proteins that modulate interactions between chromosomes, kinetochores, and microtubules in a way that is tailored to the type of cell division that will ensue. A further characteristic of shugoshins is that their association with the pericentromere appears to be modulated in response to tension. The establishment of tension between sister kinetochores causes decreased association of shugoshin with the pericentromere in several species. This has led to the idea that this relocalization of shugoshin in response to tension between sister kinetochores underlies an ancestral role of these proteins in sensing sister kinetochore biorientation. This property of shugoshins, together with their ability to associate with a variety of effector proteins, would enable essential activities to be positioned at the pericentromere only where there is a lack of tension between sister kinetochores. However, the functional relevance and molecular details underlying the response of shugoshin to tension will require clarification in future studies. Although tension is observed to alter shugoshin localization, not all of its functions are cancelled in response to tension: indeed, cohesion is largely preserved upon sister kinetochore biorientation (see above). This suggests that different pools of shugoshin are subject to distinct regulation under tension. Perhaps a larger pool of shugoshin that promotes chromosome biorientation is removed in response to tension, while a smaller, tension-independent, pool of shugoshin protects cohesin. In organisms with two shugoshins, these functions are further refined by a division of labor between them.

The importance of understanding how shugoshins operate is underscored by recent studies describing their involvement in diseases. Mice heterozygous for *Mm-SGO1* showed increased chromosome instability and susceptibility to tumors (152), and mutations in human *Hs-SGO1* have been associated with gastric and colorectal cancers (153) as well as altered heart and gut rhythm (154). As the list of roles and effector proteins for shugoshins grows longer, a detailed and molecular characterization of their functions and regulation across multiple organisms will be required to uncover conserved principles.
